# Cause-Specific Excess Mortality in Siblings of Patients Co-Infected with HIV and Hepatitis C Virus

**DOI:** 10.1371/journal.pone.0000738

**Published:** 2007-08-15

**Authors:** Ann-Brit Eg Hansen, Nicolai Lohse, Jan Gerstoft, Gitte Kronborg, Alex Laursen, Court Pedersen, Henrik Toft Sørensen, Niels Obel

**Affiliations:** 1 Department of Infectious Diseases, Odense University Hospital, Odense, Denmark; 2 University of Southern Denmark, Odense, Denmark; 3 The Danish HIV Cohort Study, Rigshospitalet, Copenhagen, Denmark; 4 Department of Clinical Epidemiology, Aarhus University Hospital, Aarhus, Denmark; 5 Department of Infectious Diseases, Rigshospitalet, Copenhagen, Denmark; 6 Department of Infectious Diseases, Hvidovre University Hospital, Copenhagen, Denmark; 7 Department of Infectious Diseases, Aarhus University Hospital Skejby, Aarhus, Denmark; 8 Department of Epidemiology, Boston University, Boston, Massachusetts, United States of America; Instituto de Pesquisa Clinica Evandro Chagas, FIOCRUZ, Brazil

## Abstract

**Background:**

Co-infection with hepatitis C in HIV-infected individuals is associated with 3- to 4-fold higher mortality among these patients' siblings, compared with siblings of mono-infected HIV-patients or population controls. This indicates that risk factors shared by family members partially account for the excess mortality of HIV/HCV-co-infected patients. We aimed to explore the causes of death contributing to the excess sibling mortality.

**Methodology and Principal Findings:**

We retrieved causes of death from the Danish National Registry of Deaths and estimated cause-specific excess mortality rates (EMR) for siblings of HIV/HCV-co-infected individuals (n = 436) and siblings of HIV mono-infected individuals (n = 1837) compared with siblings of population controls (n = 281,221). Siblings of HIV/HCV-co-infected individuals had an all-cause EMR of 3.03 (95% CI, 1.56–4.50) per 1,000 person-years, compared with siblings of matched population controls. Substance abuse-related deaths contributed most to the elevated mortality among siblings [EMR = 2.25 (1.09–3.40)] followed by unnatural deaths [EMR = 0.67 (−0.05–1.39)]. No siblings of HIV/HCV co-infected patients had a liver-related diagnosis as underlying cause of death. Siblings of HIV-mono-infected individuals had an all-cause EMR of 0.60 (0.16–1.05) compared with siblings of controls. This modest excess mortality was due to deaths from an unknown cause [EMR = 0.28 (0.07–0.48)], deaths from substance abuse [EMR = 0.19 (−0.04–0.43)], and unnatural deaths [EMR = 0.18 (−0.06–0.42)].

**Conclusions:**

HCV co-infection among HIV-infected patients was a strong marker for family-related mortality due to substance abuse and other unnatural causes. To reduce morbidity and mortality in HIV/HCV-co-infected patients, the advances in antiviral treatment of HCV should be accompanied by continued focus on interventions targeted at substance abuse-related risk factors.

## Introduction

Co-infection with HCV is a marker for poor prognosis in HIV-infected individuals. It is unclear whether the prognostic impact is caused directly by the viral disease, or whether it is an epiphenomenon of the infection [1]–[3]. It has been suggested that HCV exerts a direct pathogenic effect by compromising the immune restoration induced by highly active antiretroviral therapy (HAART) [1], [4], [5], but it has been difficult to separate the direct pathogenic effects of HCV from indirect effects of associated risk factors. In most settings, HCV infection is closely linked with intravenous drug use (IDU), which is associated with risk factors such as mental health illnesses, depression and alcohol abuse [6], [7].

In a recent attempt to uncover the impact of the indirect effects, we examined the mortality in siblings of HIV/HCV-co-infected individuals. We found three- to four-fold higher mortality among siblings of HCV/HIV-co-infected patients, compared to siblings of HIV-mono-infected individuals and siblings of population controls [8]. This indicates that risk factors shared by family members partially account for the excess mortality of HIV/HCV-co-infected patients. Given the high parenteral transmissibility of HCV, we hypothesized that HCV infection is a marker of high-risk lifestyle associated with intravenous drug use (IDU). However, the excess mortality could also be mediated through shared susceptibility to some infectious pathogens or increased prevalence of HCV infection among siblings of HIV/HCV-co-infected individuals.

To explore the excess mortality shared among family members, we used a nationwide population-based Danish cohort to compare cause-specific mortality rates, including mortality associated with drug and alcohol abuse, among three groups: siblings of HIV/HCV-co-infected individuals, siblings of HIV-mono-infected individuals, and siblings of general population controls.

## Methods

The study setting and methods used to identify siblings have been described in detail previously [8]. Briefly, we used the population-based Danish HIV Cohort Study (DHCS) [9] to identify all Danish HIV mono-infected and HIV/HCV co-infected patients treated in Danish HIV Centres since 1995. HIV treatment in Denmark is restricted to eight specialised centres, and the Danish health care system provides free tax-supported medical care, including antiretroviral treatment for HIV.

**Figure 1:**
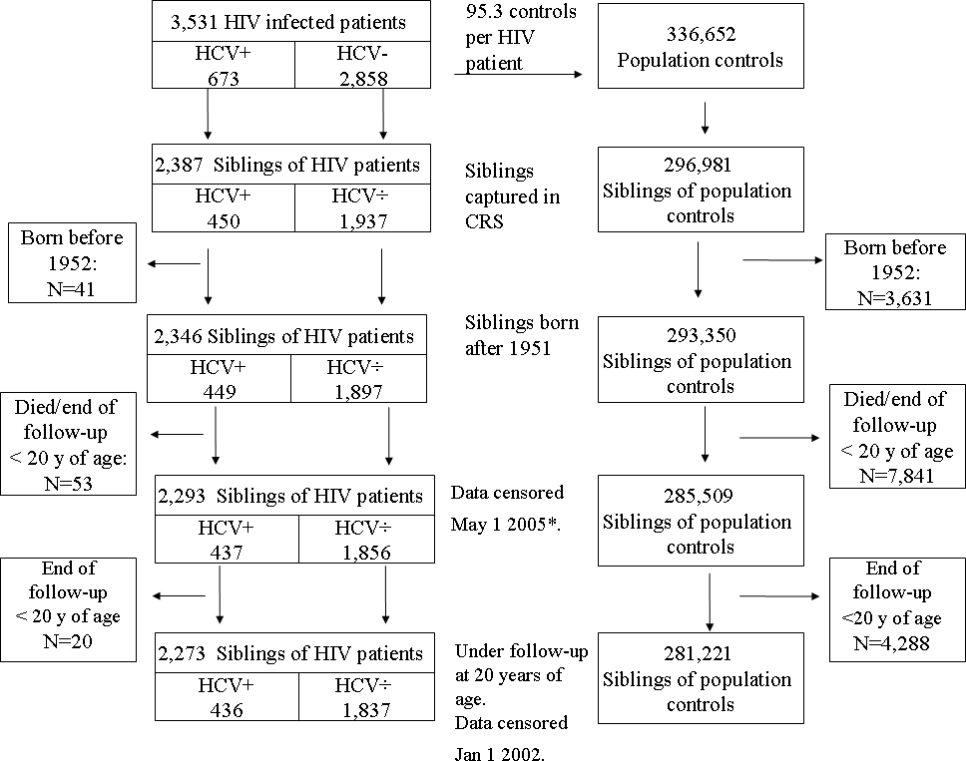
Summary of the study design. CRS, the Danish Civil Registration System; HCV, Hepatitis C virus. * Number of siblings in [8]

We identified up to 99 population controls per patient, matched by gender, age and residency from the Danish Civil Registration System (CRS). CRS is an electronic database established in 1968, which tracks vital status, migration, residency and kinship for all Danish inhabitants [10]. CRS records contain a unique 10-digit civil registration number that allows accurate record linkage with other registries. For both HIV-infected patients and population controls, we identified all full siblings born after 1951 (as registration of kinship was incomplete before this date) and obtained their dates of death or emigration from CRS. Based on the HIV patients' HCV serostatus or tests for HCV-RNA, siblings of HIV-infected individuals were classified as siblings of HIV-mono-infected individuals or siblings of HIV/HCV-co-infected individuals [8]. A flowchart of eligible individuals is displayed in Figure 1.

We used the National Registry of Deaths (NRD) [11] to document causes of death for the three sibling groups. The NRD contains information from all Danish death certificates since 1943, coded according to the Danish version of International Classification of Diseases [ICD-8 from 1972 through 1993, and ICD-10 from 1994 through 2001].

We followed the siblings from age 20 until death, emigration, or their 50^th^ birthday, whichever came first. All subjects were censored after 31 December 2001, the latest date when electronically coded causes of death were available in the NRD. In the current study, the number of siblings, person years of follow-up, and number of deaths were slightly smaller than in our earlier study of mortality among siblings of HCV/HIV-co-infected patients, in which follow up was censored on 1 May 2005 [8]. Figure 1 displays a summary of the study design for selection of siblings.

We classified causes of death into five groups, based on death certificate information:

1. Alcohol or drug abuse-related;
2. HIV (excluding those related to substance abuse);
3. Natural causes (excluding those related to HIV or substance abuse);
4. Unnatural causes (including deaths due to accidents, suicide, or homicide and excluding deaths related to substance abuse);
5. Unknown causes.

To capture deaths related to abuse of alcohol and drugs (including some prescription drugs), we used diagnoses for both the underlying (main) and contributory causes of death. The causes of death defined as substance-abuse related were: a) mental disorders associated with alcohol or drug dependence (ICD-8: 291, 303, or 304. ICD-10: F10-F19); b) toxic effect of/poisoning by alcohol (ICD-8: E860, N979, or N980. ICD-10: X45, T51, or Y15); c) non-mental diseases caused by alcohol (ICD-8: 5710, 5711, or 5771. ICD-10: G312, G621, G721, I426, K292, K70, or K860); and d) toxic effect of/poisoning by specified psychoactive drugs [accidental, suicidal or undermined whether accidentally or purposely inflicted] (ICD-8: E8530, E8540, or, E950 or E980 in combination with N9650, N9780, or N9790. ICD-10: X41, X42, or X60-X64 in combination with T40, Y11, Y12) [12]. For all other causes (group 2-5), only the underlying cause of death was used. In the Danish versions of ICD-8 and ICD-10, deaths with HIV as the underlying cause are coded as 0797 and B20-B24, respectively.

Among individuals with HIV as the underlying cause of death, the route of infection was identified from DHCS records. Deaths in those who were intravenous drug users were reclassified as a IDU-related death in a supplementary analysis, as proposed by Schulz-Schaeffer *et al.* [13]. We also performed a supplementary analysis of deaths with HCV infection or other liver diseases as the underlying cause of death.

For the three groups of siblings, we first computed total and cause-specific mortality rates (MR) by person-year analyses and used exact poisson confidence intervals. We then calculated total and cause-specific absolute excess mortality rates (EMR), comparing siblings of HIV-mono-infected and HIV/HCV-co-infected patients with siblings of population controls (the reference group). We calculated absolute EMR as the risk difference between two groups as described by Rothman [14] i.e. by subtracting the MR of siblings of population controls from the MR for siblings of each of the other two groups. We calculated 95% confidence intervals for the EMRs by using the standard error of the rate difference as described by Rothman [14], [15]. We used Stata software, version 9.2 (StataCorp, College Station, TX, USA) for statistical analyses. The study was approved by the Danish Data Protection Agency.

## Results

### Patients, population controls and their siblings

As of 1 May 2005, DHCS included 4,261 Danish residents who were older than 16 years at the time of HIV diagnosis. We excluded 271 patients not living in Denmark at time of HIV diagnosis and 459 patients who were never tested for HCV. Of the remaining 3,531 patients, 673 had HIV/HCV co-infection and 2858 were HIV-mono-infected. Owing to an insufficient number of eligible controls in some municipalities, the study included a mean of 95.3 population controls per HIV patient, yielding 336,652 matched controls. The HIV/HCV co-infected patients had 436 siblings, the HIV mono-infected patients had 1,837 siblings, and the population controls had 281,221 siblings. Nine families included two siblings with HIV. Hence, 18 persons were included as both HIV patients and siblings of HIV patients.

### Mortality rates of siblings

The 436 siblings of HIV/HCV-co-infected individuals contributed 7,199 person-years of follow-up (PYR), followed from age 20 years. During this time we observed 29 deaths [MR = 4.03 per 1,000 PYR] (Table 1). Eighteen deaths were substance abuse-related [MR = 2.50 per 1,000 PYR]. The 1,837 siblings of HIV-mono-infected individuals contributed 31,232 PYR and 50 deaths [MR = 1.60 per 1,000 PYR], with 14 deaths [MR = 0.45 per 1,000 PYR] attributable to substance abuse. Finally, the 281,221 siblings of population controls contributed 4,590,754 PYR and 4,588 deaths [MR = 1.00 per 1,000 PYR], of which 1,169 deaths [MR 0.25 per 1,000 PYR] were substance abuse-related.

**Table 1:** Total and cause-specific MR and excess MR among the three groups of siblings.

Causes of deaths in sibling groups	Number of deaths	Person-years	MR per 1000 PY (95% CI)	Excess mortality rates (95% CI)
**Total**				
Control	4588	4,590,754	1.00 (0.97–1.03)	
HIV	50	31,232	1.60 (1.19–2.11)	0.60 (0.16–1.05)
HIV/HCV	29	7,199	4.03 (2.70–5.79)	3.03 (1.56–4.50)
**Substance abuse**				
Control	1169	4,590,754	0.25 (0.24–0.27)	
HIV	14	31,232	0.45 (0.25–0.75)	0.19 (−0.04–0.43)
HIV/HCV	18	7,199	2.50 (1.48–3.95)	2.25 (1.09–3.40)
**Unnatural causes**				
Control	1,381*	4,590,754	0.30 (0.29–0.32)	
HIV	15**	31,232	0.48 (0.27–0.79)	0.18 (−0.06–0.42)
HIV/HCV	7***	7,199	0.97 (0.39–2.00)	0.67 (−0.05–1.39)
**HIV**				
Control	137	4,590,754	0.03 (0.03–0.04)	
HIV	2	31,232	0.06 (0.01–0.23)	0.03 (−0.05–0.12)
HIV/HCV	3	7,199	0.42 (0.09–1.22)	0.39 (−0.08–0.86)
**Natural causes**				
Control	1,547	4,590,754	0.34 (0.32–0.35)	
HIV	8	31,232	0.26 (0.11–0.50)	−0.08 (−0.26–0.10)
HIV/HCV	1	7,199	0.14 (0.00–0.77)	−0.20 (−0.47–0.07)
**Unknown mode/cause of death**				
Control	354	4,590,754	0.08 (0.07–0.09)	
HIV	11	31,232	0.35 (0.18–0.63)	0.28 (0.07–0.48)
HIV/HCV	0	7,199	0 †	−0.08†

* 649 accidents, 618 suicides, 69 homicides, 45 injuries undetermined whether accidentally or purposely inflicted

** 6 suicides, 9 accidents,

*** 3 suicides, 3 accidents, 1 injury undetermined whether accidentally or purposely inflicted

† Because there were no deaths from unknown causes in the HIV/HCV group, we were not able to calculate 95% confidence intervals (CI).

### Excess mortality rates of siblings

Total and cause-specific MR and EMR with 95% confidence intervals for all groups of siblings are displayed in Table 1. The all-cause EMR for siblings of HIV/HCV-co-infected individuals was 3.03 per 1,000 PYR, compared with siblings of population controls. This excess mortality was mainly caused by substance abuse-related deaths (EMR = 2.25 per 1000 PYR) and to a lesser extent by unnatural deaths (EMR = 0.67 per 1,000 PYR) and by deaths with HIV as the underlying cause (EMR = 0.39 per 1,000 PYR).

Siblings of HIV-mono-infected individuals had an all-cause EMR of 0.60 per 1,000 PYR compared with siblings of population controls. This small excess mortality was due to deaths from an unknown cause (EMR = 0.28 per 1,000 PYR), deaths from substance abuse (EMR = 0.19 per 1,000 PYR), and unnatural deaths (EMR = 0.18 per 1,000 PYR).

### Substance abuse in HIV-related deaths

Three siblings of HIV/HCV-co-infected individuals and 137 control siblings had HIV as their underlying cause of death. Of these, two siblings of HIV/HCV-co-infected individuals and four control siblings were registered in DHCS with IDU as the route of HIV transmission. When these deaths were counted as substance abuse-related deaths, the cause-specific mortality rate for siblings of HIV/HCV-co-infected individuals increased from 2.50 to 2.78 per 1,000 PYR, and the EMR increased from 2.25 to 2.52 per 1,000 PYR. The substance abuse-related mortality rate for controls changed slightly from 0.25 to 0.26 per 1,000 PYR.

### HCV-related deaths

No siblings of HIV/HCV-co-infected patients had acute or chronic HCV infection or any other liver-related diagnosis as the underlying cause of death.

## Discussion

Our nationwide study showed that the increased mortality in siblings of HIV/HCV-co-infected patients is mainly caused by alcohol and drug abuse-related deaths. The study did not provide evidence that hepatitis C infection or increased inherited susceptibility to other infectious diseases contribute to the excess mortality in siblings of HIV/HCV co-infected patients.

These results lend credence to the hypothesis that excess mortality in HIV/HCV-co-infected patients, compared to HIV mono-infected patients, partly stems from factors other than the HCV disease itself, and a large part of the increased mortality in HIV/HCV co-infected patients - compared to HIV mono-infected patients - may be caused by similar family-associated and lifestyle-related factors. In the co-infected patients, substance abuse and associated risk behaviors may directly affect mortality. Indirectly, mortality in HIV/HCV-co-infected patients may be elevated by suboptimal health-seeking behavior, poor adherence to medical treatment, drug- or alcohol-related comorbidity, or health care providers' inattention to these patients. In addition, clinicians may choose to postpone antiretroviral therapy in HIV/HCV-co-infected patients due to concerns about behavioral factors or liver toxicity, despite evidence that early treatment initiation is beneficial for these patients [16].

The mechanisms underlying clustering of substance abuse-related deaths in families of HIV/HCV-co-infected individuals require better understanding. A likely explanation is that the common childhood and environment among siblings lead to a similar high risk of substance abuse. However, adoption and twin studies have shown that in addition to family environment, there is a strong genetic contribution to a familial predisposition for substance abuse [17], [18]. In any case, familial clustering of the detrimental effects of substance abuse underscores the need for adequate control groups, and/or extensive control of confounding when effects of HCV are investigated in epidemiological settings.

Further evaluation is also needed to determine whether these family-related risk factors have the same influence on prognosis in HCV-mono-infected patients, because HIV/HCV-co-infected individuals may represent a more vulnerable subgroup than HCV-mono-infected individuals. In line with our results, a large population-based study has recently demonstrated that young people with HCV infection have higher mortality from continued drug uses than from the HCV infection [19].

Our study had a number of strengths and limitations. Use of longitudinal population-based Danish registries permitted complete long-term follow-up with minimal selection bias, while the matched selection of siblings assured comparability of groups in terms of birth year and residency [8]. Because it has been shown that relying exclusively on underlying cause of death leads to underestimation of alcohol- and drug-related mortality, we used both underlying and contributory causes [12], [13] to specify these causes of death. Still, death certificate data are not entirely accurate [20], and potential differential misclassification could lead to underestimation of the contribution of substance abuse to the category of unnatural deaths [13], [21]. We could not assess mode of HIV infection in all HIV-related deaths among siblings of population controls. However, the resulting bias is probably negligible, since both the MR of HIV-related deaths and the proportion of HIV infections acquired by IDU among those with HIV-related deaths were small in this group. Finally, it is important to note that the results of this study may not be generalizable to settings where HIV/HCV-co-infected patients do not have IDU as the main route of transmission, for example Africa [22].

In conclusion, HCV co-infection among HIV-infected patients seemed to be a strong marker for family-related mortality due to substance abuse. In order to reduce morbidity and mortality in HIV/HCV co-infected patients, advances in antiviral treatment of HCV should be accompanied by interventions targeted at substance abuse and associated risk factors.
